# Developing interpretable machine learning-Shapley additive explanations model for unconfined compressive strength of cohesive soils stabilized with geopolymer

**DOI:** 10.1371/journal.pone.0286950

**Published:** 2023-06-08

**Authors:** Anh Quan Ngo, Linh Quy Nguyen, Van Quan Tran

**Affiliations:** 1 Hydraulic Construction Institute—Vietnam Academy For Water Resources, Hanoi, Vietnam; 2 University of Transport Technology, Thanh Xuan District, Hanoi, Vietnam; Middle East Technical University, TURKEY

## Abstract

This paper seeks to develop an interpretable Machine Learning (ML) model for predicting the unconfined compressive strength (UCS) of cohesive soils stabilized with geopolymer at 28 days. Four models including Random Forest (RF), Artificial Neuron Network (ANN), Extreme Gradient Boosting (XGB), and Gradient Boosting (GB) are built. The database consists of 282 samples collected from the literature with three different types of cohesive soil stabilized with three geopolymer categories including Slag-based geopolymer cement, alkali-activated fly ash geopolymer and slag/fly ash-based geopolymer cement. The optimal model is selected by comparing their performances with each other. The values of hyperparameters are tuned by Particle Swarm Optimization (PSO) algorithm and K-Fold Cross Validation. Statistical indicators show the superior performance of the ANN model with three metrics performance such as coefficient of determination R^2^ = 0.9808, Root Mean Square Error RMSE = 0.8808 MPa and Mean Absolute Error MAE = 0.6344 MPa. In addition, a sensitivity analysis was performed to determine the influence of different input parameters on the UCS of cohesive soils stabilized with geopolymer. The order of feature effect can be ordered in descending order using the Shapley additive explanations (SHAP) value as follows: Ground granulated blast slag content (GGBFS) > Liquid limit (LL) > Alkali/Binder ratio (A/B) > Molarity (M) > Fly ash content (FA) > Na/Al > Si/Al. The ANN model can obtain the best accuracy using these seven inputs. LL has a negative correlation with the growth of unconfined compressive strength, whereas GGBFS has a positive correlation.

## 1. Introduction

Currently, the materials used to fill the roadbed, and warehouse foundation often use granular soil. With the current huge number of projects, the source of loose granular materials such as sand and embankment are in short supply compared to the demand. At the same time, sand mining also causes risks with serious impacts on the environment if there is no suitable plan and scale. Cohesive soil is considered as an alternative material to be used to compensate for this depletion of the source material. Due to the unsuitable geotechnical properties of cohesive soils, many studies have been carried out to improve this such as the use of stabilizers: lime, fly ash… The study of geopolymer technology to improve cohesive soil has scientific and practical significance. Geopolymer is the word used to refer to inorganic materials synthesized from Aluminosilicate. The raw material system for making geopolymer materials includes two main components: starting materials and alkaline chemical active ingredients.

Geopolymer materials are investigated to create environmentally friendly products, taking advantage of industrial wastes with high usability [1–3]. In the world, the applications of geopolymer are very wide in acid-resistant cement, unburnt brick, fast-curing cement, and fireproof material… Geopolymer technology is very interesting and well researched in the world with development prospects. Rios et al. [4] provide some findings from a geopolymer-stabilized soil experiment in Colombia. 16 specimens made with various concentrations of fly ash, soil, and alkaline solutions were analyzed using UCS and stiffness tests. The addition of 0% to 20% geopolymer content to peat soil was the subject of Zain et al. investigation [5]. To estimate the resilience modulus model as a function of deviator stresses and radial stresses, the resilience modulus was measured using a cyclic triaxial test. According to the test results, increasing radial stresses did not always result in increasing modulus robust; on the other hand, increasing deviator stresses caused a reduction in modulus resilient. The rise in robust modulus value was not significantly impacted by the addition of geopolymer to peat soil. The research of Abdullah and Shahin [6] focuses on the geo-mechanical properties of a relatively novel clay-binder combination made from fly ash activated by alkali, which results in a geopolymer that hardens to cement soil. Based on the results, geopolymer-treated clay specimens have stronger mechanical properties untreated clay specimens and shown by unconfined compressive strength (UCS) test and Consolidated Undrained (CU) test. The unconfined compressive strength might increase by up to six times depending on the geopolymer content and curing duration. Confining pressure is another element that raises the stiffness and, as a result, the undrained peak strength of clay treated with geopolymer unconfined compressive strength. The engineering characteristics of cohesive soil stabilized by geopolymers depend on some factors, such as the nonhomogeneous nature of their constituent parts, the inherent differences in properties of various elements, and occasionally, the dual or conflicting effects of some ingredients on the overall performance of the soil. Hence, in order to properly utilize these materials in diverse constructions, a good knowledge of such complicated behavior is required. Machine Learning (ML) models have been successfully used to investigate different complex problems of civil such as construction building materials [7–10], geotechnical problem [11].

In regard of ML model applications in geopolymer stabilized soil, some investigations are performed in recent times. Firstly, Mozumder and Laskar [12] used the database containing 282 samples and eight input variables such as liquid limit (LL), plastic index (PI), content of ground granulated blast-furnace slag (GGBFS), content of fly ash (FA), molarity of NaOH concentration (M), alkaline content/binder content ratio (A/B), atomic number of Na to atomic number of Al (Na/Al), and atomic number of Si to atomic number of Al (Si/Al) as input parameters for developing Artificial Neural Network (ANN) model including multi-layer perception (MLP) feed-forward network and Bayesian Regularization back propagation training technique in predicting 28 days UCS of geopolymer stabilized clayey soil. The ANN model proposed by Mozumder and Laskar [12] has the performance metric as coefficient of determination R^2^ = 0.9643 for testing dataset. Generating a new database from the database of Mozumder and Laskar [12], the new database contains 213 samples and the same the same input variables without the input FA content. Basing this new database, Mozumder et al. [13] predicted UCS of geopolymer stabilized soil using the Support Vector Machine (SVM) model with performance metric R^2^ = 0.9801 for testing dataset. Javdanian and Lee [14] developed a hybrid Machine Learning model included neuro-fuzzy (NF)-group method of data handling (GMDH) and particle swarm optimization (PSO) abbreviated (NF-GMDH-PSO) in predicting UCS of stabilized cohesive soils using geopolymers with the performance metric such as R^2^ = 0.971, Mean Absolute Error MAE = 0.231 MPa and Root Mean Square Error RMSE = 0.401 MPa. The efficiency of the particle swarm optimization (PSO) algorithm in predicting UCS of geopolymer stabilized expansive blended clays was also investigated by Nagaraju and Prasad [15]. Soleimani et al. [16] used the original database consisting of 282 samples and 8 input variables for building multi-gen genetic programming (MGGP) model for estimating UCS of geopolymer stabilized soil, the performance of this model is high with R^2^ = 0.942 and MAE = 1.071 MPa for testing dataset. Recently, Zeini et al. [17] used a popular Machine Learning algorithm named Random Forest (RF) in strength prediction of geopolymer stabilized clayey soil, the authors used the original database, the performance of the ML model is evaluated by R^2^ = 0.9757 and RMSE = 0.9815 MPa for testing dataset.

It can be seen that 5 out of 6 studies mentioned above use the same dataset origin on geopolymer stabilized soil, as well as use the same 8 input variables including LL, PI, GGBFS, FA, M, A/B, Na/Al, and Si/Al. These variables represent the properties of the soil to be reinforced (LL, PI), the composition of the geopolymer including binder content B (GGBFS+FA), alkaline activator content A/B, Na/Al, Si/Al (NaOH and Na_2_SiO_3_), molar concentration M of the NaOH activator. Therefore, these 8 variables can be considered as necessary input variables for setting up a Machine Learning model for predicting the UCS of geopolymer stabilized soil.

However, the above studies have not quantified the influence of each variable on the value of UCS by machine learning models. This is of great significance in the selection and design of geopolymer compositions for soil reinforcement. Therefore, in order to quantify each factor affecting UCS, the goal presented in this paper will focus on improving the performance of the machine learning model in predicting the UCS of geopolymer stabilized soil based on the UCS. The eight input variables mentioned above include LL, PI, GGBFS, FA, M, A/B, Na/Al, and Si/Al. The models with the highest performance UCS prediction will be explained by Shapley Additive Explanation (SHAP) technique including global SHAP value and local SHAP value to quantify the influence of input variables on UCS, as well as on the performance of the machine learning model. The SHAP interpretation of ML models also helps to improve the UCS prediction performance of the machine learning model. To help achieve the research goal, 4 popular machine learning algorithms available in the open libraries of the Python programming language will be used including Artificial Neuron Network (ANN), Random Forest (RF), Gradient Boosting (GB) and Extreme Gradient Boosting (XGB). The hyperparameter values of these 4 models will be tuned using the Particle Swarm Optimization (PSO) algorithm. The performance of 4 ML models will be evaluated through 3 metrics including R^2^, RMSE and MAE.

## 2. Database analysis for machine learning model

The study used a database collected from Mozumder and Laskar’s study [12]. From three different type of soils represented by Liquid limit (LL) and Plasticity index (PI), 282 UCS samples of cohesive soils stabilized with geopolymer at 28 days were collected. The mechanical properties and the ratio of the binder compounds are 8 input variables including: Liquid limit LL (%), Plastic index PI (%), Ground granulated blast slag content GGBFS (%), Fly ash content FA (%), Molarity of NaOH M (mol/l), Alkali/Binder ratio A/B, Na/Al, Si/Al, along with an output variable UCS of cohesive soils stabilized with geopolymer. As a proportion of the dry weight of the soil solids, the amount of binder ranged between 4–50% for GGBFS, 4–20% for FA, and a combination of GGBFS + FA. In the investigation of Mozumder and Laskar [12], alkali solutions with molar concentrations of 4 M, 8 M, 10 M, 12 M, and 14.5 M were utilized. Alkali solution to binder by weight (A/B) ratio was selected as 0.45, 0.65, and 0.85. To remove air gaps, samples were manually crushed in PVC molds with dimensions of 38 mm in diameter and 76 mm in height to the limit of plastic consistency. Since the samples were manually compacted, it was discovered that necessary workability for homogenous compaction was attainable at a consistency of plastic limit. The produced samples were placed in molds and left in the lab for 24 hours before being continually cured in water for 28 days. Samples were cured, air dried at room temperature for an hour, and then tested. The specimens’ 28 day UCS test was performed in accordance with Indian Standard: 2720 [18].

**Fig 1:**
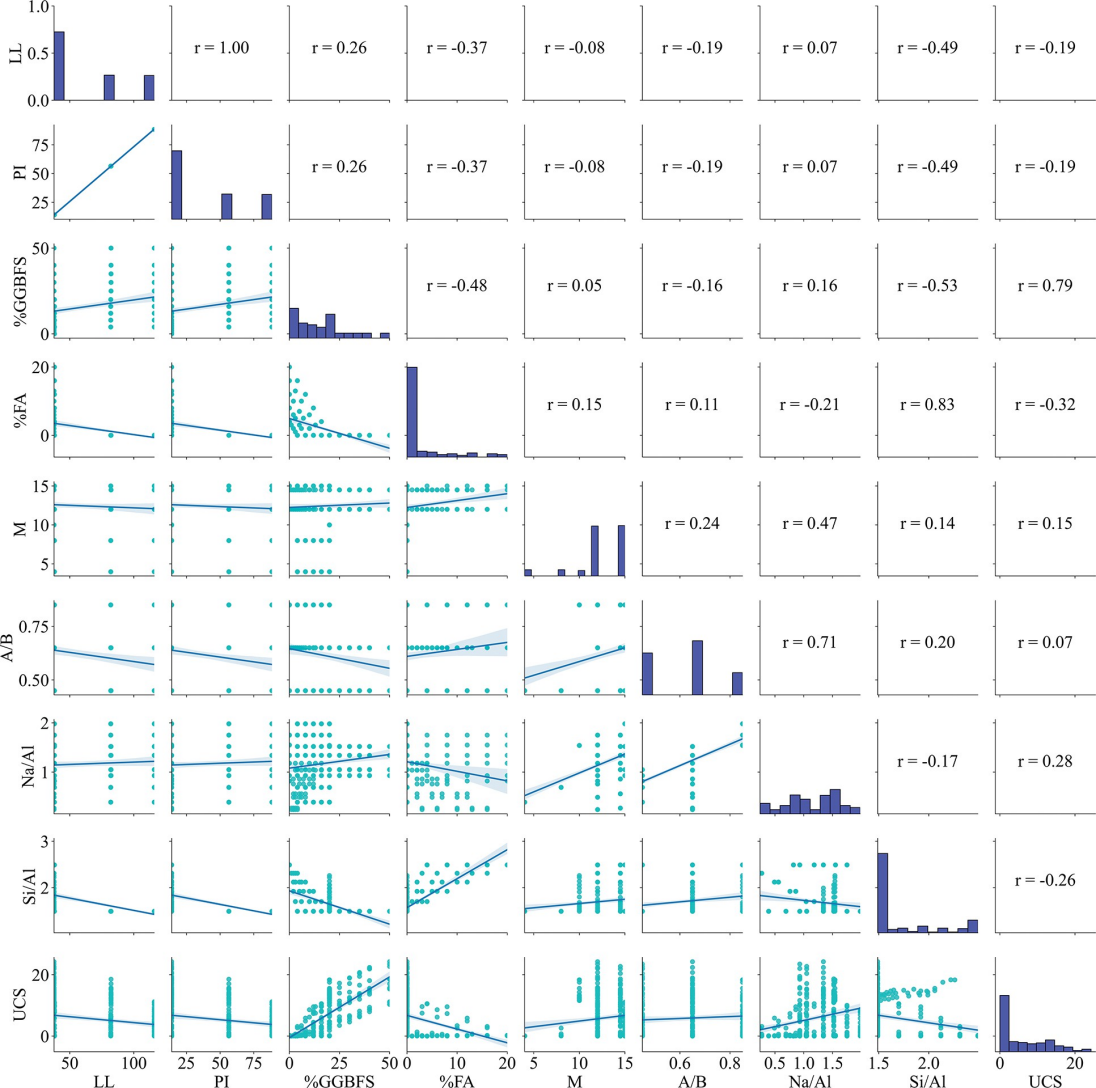
Correlation plot of input variables and output variables of cohesive soils stabilized with geopolymer.

Fig 1 depicts the data distribution and correlation analysis of the input and output variables in this study. As shown in the figure, the variables are insignificantly correlated. The highest linear correlation coefficient between GGBFS and UCS is 0.79. The inputs are relatively bumpy against each other. The samples of the training part will greatly affect the accuracy of the model after training, the samples are randomly taken to divide into two parts of the data used for training and testing. Table 1 provides a rough summary of all the data samples collected, that contains Minimal value, the first quartile (Q1) Q_25%_, the second quartile (Q2) Median, Mean value, the third quartile (Q3) Q_75%_, Maximal value, Standard deviation value Std. 70% of the samples (197 samples) randomly selected to be used as training data for machine learning models are listed in Table 2. The remaining 85 data samples used for model testing are summarized. Statistical details of 85 samples for testing dataset are described in Table 3.

**Table 1:** Statistical description of all dataset.

	Min	Q_25%_	Median	Mean	Q_75%_	Max	Std
LL (%)	37.70	37.70	38.00	63.59	82.20	116.00	32.17
PI (%)	14.07	14.07	14.07	38.65	56.46	88.46	30.65
GGBGS content (%)	0.00	4.25	16.00	15.94	20.00	50.00	12.93
FA content (%)	0.00	0.00	0.00	2.13	0.00	20.00	4.67
M (mol/l)	4.00	12.00	12.00	12.43	14.50	15.00	2.73
Alkali/Binder ratio A/B	0.45	0.45	0.65	0.62	0.65	0.85	0.14
Na/Al	0.24	0.93	1.18	1.17	1.52	1.98	0.44
Si/Al	1.49	1.49	1.49	1.70	1.89	2.49	0.35
UCS (MPa)	0.00	0.12	2.96	5.79	10.94	24.26	6.50

**Table 2:** Statistical description of training dataset.

	Min	Q_25%_	Median	Mean	Q_75%_	Max	Std
LL (%)	37.70	37.70	38.00	61.71	82.00	116.00	31.45
PI (%)	14.07	14.07	14.07	36.86	56.46	88.46	29.97
GGBGS content (%)	0.00	4.00	16.00	15.57	20.00	50.00	12.62
FA content (%)	0.00	0.00	0.00	2.21	1.00	20.00	4.85
M (mol/l)	4.00	12.00	12.00	12.40	14.50	15.00	2.70
Alkali/Binder ratio A/B	0.45	0.45	0.65	0.61	0.65	0.85	0.14
Na/Al	0.24	0.93	1.18	1.16	1.52	1.98	0.44
Si/Al	1.49	1.49	1.49	1.70	1.75	2.49	0.35
UCS (MPa)	0.00	0.11	2.91	5.75	10.79	24.26	6.55

**Table 3:** Statistical description of testing dataset.

	Min	Q_25%_	Median	Mean	Q_75%_	Max	Std
LL (%)	37.70	37.70	38.00	67.93	116.00	116.00	33.57
PI (%)	14.07	14.07	14.07	42.80	88.46	88.46	31.95
GGBGS content (%)	0.00	6.00	12.00	16.80	20.00	50.00	13.65
FA content (%)	0.00	0.00	0.00	1.94	0.00	16.00	4.26
M (mol/l)	4.00	12.00	12.00	12.48	14.50	15.00	2.81
Alkali/Binder ratio A/B	0.45	0.45	0.65	0.63	0.65	0.85	0.14
Na/Al	0.24	0.93	1.34	1.20	1.52	1.98	0.44
Si/Al	1.49	1.49	1.49	1.70	1.92	2.49	0.35
UCS (MPa)	0.00	0.17	3.14	5.86	12.16	20.72	6.40

## 3. Machine learning theory

### 3.1 Extreme Gradient Boosting algorithm

XGBoost, which stands for Extreme Gradient Boosting [19], is a well-liked and effective open-source version of the technique of the gradient-boosted tree. The XGBoost method combines the predictions of weak learners using additional techniques to create an effective learning model [20]. The XGBoost classifier not only performs well and quickly, but it also avoids overfitting and makes the most of available computer power. These benefits are brought about by the goal functions’ simplicity, which makes it possible to combine regularization and prediction terms and allows for the possibility of parallel execution during the training stage. Regarding its methodology, the first learner is trained to the full dataset. The errors made by the first pupil are subsequently made available to the second learner. The final prediction model is then produced by combining the predictions of all learners, and this procedure is continued until the stopping condition is satisfied.


$$f_{i}^{\left(p\right)}=\sum_{m‐1}^{p}f(x_{i}){=f}_{i}^{\left(p‐1\right)}{+f}_{p}(x_{i})$$ (1)

The training data *x*_*i*_ is used to predict the target $f_{i}^{\left(p\right)}$ at step *p* and $f_{i}^{\left(p‐1\right)}$ at step *(p-1)*.

The objective function of the model is presented by Formula (2)

$$Obj^{\left(t\right)}=\sum_{k=1}^{m}L(\hat{f},f)+\sum_{k=1}^{p}\alpha (f_{i})$$ (2)

where n is the number of observations, L denotes the loss function, and α denotes the regularization function.


$$\alpha (f)=\beta T+\frac{1}{2}{\delta \mu }^{2}$$ (3)

### 3.2 Gradient boosting algorithm

For regression and classification problems, gradient boosting (GB) is a machine learning approach that generates a model in the form of a group of weak prediction models, often decision trees [21]. Similar to previous boosting techniques, it constructs the model in stages, but it generalizes them by enabling the optimization of any differentiable loss function [22].

Three essential processes are involved in gradient boosting. Optimizing a loss function is the first step that must be taken. A differentiable loss function is required. How well a machine learning model matches the data for phenomena is measured by a loss function. Depending on each issue, a different loss function may be utilized. The weak learner is used for the second phase. A decision tree serves as the weak learner in gradient boosters. Regression trees that produce actual values for splits and whose output can be added together are specifically employed, allowing for the addition of output from successive models to correct residuals in predictions from the prior iteration. Although the methods for classification problems and regression issues differ, they both employ the same strategy for classifying the data. Decision trees used in regression is that method. The third phase involves adding together a lot of weak learners. One by one, decision trees are added. While adding trees, a gradient descent approach is utilized to reduce loss. The gradient component of gradient boosters is that. Instead of using the parameters of the weak models, the gradient descent optimization is applied on the model’s output.

In slope enhancement 0<m<p times, suppose there is an imperfect model *fi* initially. The gradient enhancement algorithm does not change it but builds a new model $f_{p}(x)=f_{p‐1}(x_{i})+f(x_{i})$ by adding an estimator f to improve the efficiency of the model Ft. GB to find the best f as follows:

$$f_{p}(x_{i}){=f}_{p‐1}(x_{i})+f(x_{i})$$ (4)

equivalently:

$$f(x_{i})=f_{p}(x_{i})-f_{p‐1}(x_{i})$$ (5)

Thus, the gradient boosting algorithm fits f with the residual *f*_*p*_(*x*_*i*_)−*f*_*p-1*_(*x*_*i*_). Like other boosting variants, *f*_*p*_ modifies its predecessor *f*_*p-1*_. Observing that the residual *f*_*p*_(*x*_*i*_) is the negative gradient direction of the loss function 1/*f*_*p*_(*x*_*i*_), it can be generalized to other loss functions that are not a squared error (classification or ranking problems). That is, the gradient boosting algorithm is a gradient descent algorithm that can be generalized only by changing the loss function and the gradient.

### 3.3 Random forest algorithm

An ensemble machine-learning technique called a random forest (RF) is utilized for classification and regression problems [23]. It is made up of several decision trees, each of which was trained using a different random subset of the data. The average or majority vote of all the individual decision trees’ outputs constitutes the random forest’s ultimate result. RF’s primary principle is that the method can decrease overfitting and raise the overall accuracy of the model by training numerous decision trees on various subsets of the data and then averaging or voting their results. To decorrelate the trees and further prevent overfitting, a level of randomization is introduced by the unpredictability in the subsets of data and the characteristics utilized in each tree. In many different applications, including speech recognition, object identification, and picture classification, the random forest has shown to be a potent algorithm. While aiming to prevent overfitting from occurring when creating a decision tree, random forest performs wonderfully. It also functions well when the data includes distinct elements. Other algorithms, such as strategic relapse, can outperform the random forest in terms of numerical aspects, but when it comes to deciding based on the situation, the RF is the best option.

RF provides two types of node significance metrics:

- Mean Decrease Impurity (MDI) based on the Gini index:

$$mx_{i}=v_{i}C_{i}‐v_{lefti}C_{lefti}‐v_{righti}C_{righti}$$ (6)

- Mean Decrease Accuracy (MDA) based on Out-of-Bag data (OOB)

$$mx_{i}=\frac{1}{p}\sum_{m=1}^{p}x_{i}^{m}$$ (7)

The following formula is used to determine the significance of each characteristic on the decision tree:

$$f(x_{i})=\frac{\sum_{i}mx_{i}}{\sum_{p}mx_{p}}$$ (8)

### 3.4 Artificial Neural Network algorithm

The concept behind Artificial Neural Network (ANN) algorithm is based on the premise that by simulating the real neurons and dendrites in the human brain [24]. A neuron is the fundamental building block of the incredibly potent neural network found in the human brain. The brain has billions of neurons, each of which is intricately related to the others. Each neuron functions communicates with the surrounding neurons and transmits the information that is required. The abundance of these fundamental parts and the numerous connections that exist between them are what give the human mind its strength. In essence, the brain steadily picks up new skills over time by experiencing new things. While it’s true that computers can readily accomplish sophisticated mathematical computations, they struggle to perform tasks like pattern recognition and remembering prior patterns in order to take future actions. Such pattern recognition and face recognition issues are simple for the human brain to handle. So, using artificial neural networks, which are nothing more than a collection of algorithms, researchers tried to simulate this kind of behavior. Hence, may say that artificial neural networks are a group of programs that attempt to emulate the behavior of the brain’s neural network. The objective is to create an artificial neural network that can learn over time, much like a human brain does, and recognize various patterns using the information that it has acquired via learning.

Depending on the application, the artificial neural network might include anywhere from a few tens to hundreds of thousands of neurons. These neurons are organized in many layers that are interconnected. A network will respond by recognizing input from the outside world that is received by an input layer. Depending on how the network analyzes the input and learns about it, there are output units that produce output following the applied input units. Then, between the input and output layers, there are some concealed units. A weight, which can be positive or negative, is a number that illustrates the links between two units. A weight is multiplied by each input. The strength of the weight’s input message determines how much weight there is. Each neuron node computes a weighted sum, which is then applied to an activation function to produce the outputs. Feed-forward neural networks are those in which the output from one layer is utilized as the input for the following layer. A network is given known inputs and the intended outputs that go along with them. The output that results is then compared to the known output; if there is a discrepancy, it is reported, and the network adjusts as necessary. This procedure is repeated until the ideal distribution of weights is achieved. When the learning process is over, the neural network may even spot brand-new patterns that are similar to previously learned ones.

The accumulation of the input data *xi* and the weights *wi* plus the bias *b* produces the result *z*:

$$z=\sum_{i=1}^{m}w_{i}x_{i}+b$$ (9)

Processing z through the activation function finally obtains the output ${\hat{f}}_{i}$ of the neuron. Commonly used activation functions:

Sigmoid:

$$f(x_{i})=\frac{1}{1+e^{-(x_{i})}}$$ (10)

Tanh:

$$f(x_{i})=\frac{e^{\left(x_{i}\right)}-e^{-(x_{i})}}{e^{\left(x_{i}\right)}+e^{-(x_{i})}}$$ (11)

ReLU:

$$f(x_{i})=\max(0,x_{i})$$ (12)

### 3.5 Hyperparameters tuned with Particle Swarm Optimizations

The meta-heuristic optimization technique known as Particle Swarm Optimization (PSO) is capable of solving a wide range of optimization issues [25, 26]. The cost function’s differentiability is one example of a rigorous assumption that it does not make. It is frequently used on joint optimization issues using a global single-cost function. PSO employs stochastic perturbations to find the cost function’s optimal point. It produces a large number of randomly dispersed particles, which move randomly until convergence. While traveling, they share their individual bests. The direction in which the particles move in is determined by a combination of their previous velocity, personal best, and world best [27]. Including prior velocity assures dependence on earlier calculations, moving toward the global best promotes convergence to the ideal, and including personal best promotes a variety of searches. When the optimization process continues, convergence happens as personal and global bests approach one another.

Determine the position of each particle when considering the objective function:

$$c=f(x_{i},y_{i})=\sin x^{2}+\sin y^{2}+\sin x\sin y$$ (13)

The velocity of the particles is determined by the formula:

$$V_{i}=aV_{i-1}+d_{1}U_{1}(P_{b1}-P_{i})+d_{2}U_{2}(g_{b}-P_{i})$$ (14)

New position of the particles:

$$P_{i+1}=P_{i}+V_{i+1}$$ (15)

### 3.6 K-fold validation technique

**Fig 2:**
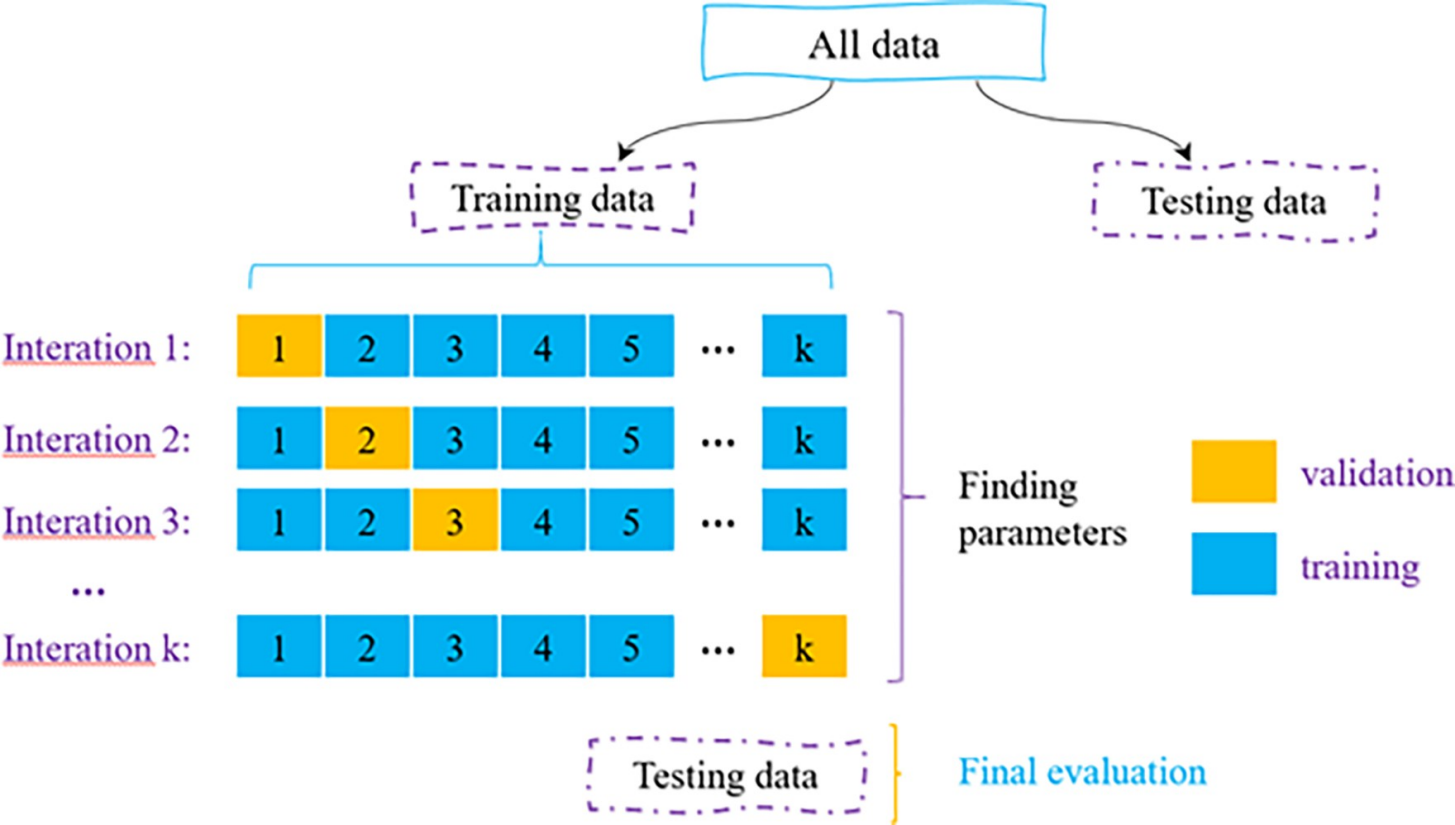
K-fold cross validation diagram.

Upon training, the model’s performance is assessed using a test procedure in which the classification result of the network is calculated using fresh data supplied into the input layer. As a result, the available dataset is first split into two sections that will be utilized separately for training and testing. For the training/test data division, it is usual practice to randomly divide the data into two halves. If a portion of the data is only used for the test, this may not produce a trustworthy evaluation of the network for a short dataset. Moreover, training or test datasets with various ratios of output classes may result from random division. Particularly with datasets with unbalanced output classes, this occurs. Divide the data into k sets and use k-1 for training and 1 for cross-validation when using k-fold cross-validation. Leave-one-out cross-validation is being used here. The data is divided into k sets (k>p), k-p sets are used to train the data, and p sets are utilized for cross-validation in leave-p-out cross-validation. Fig 2 describes briefly the K-Fold Cross Validation technique.

### 3.7 Performance evaluation of machine learning model

A model’s behavior, tasks, and performance are all tracked using performance metrics. This information should come in the form of measurements of the necessary data within a range, enabling the formation of a foundation for the accomplishment of the overall study objectives. Indicators of performance should be monitored so that changes can be made to processes to achieve optimum performance. In this study, three performance indicators coefficient of determination (R^2^), the Root Mean Square Error (RMSE), and the Mean Absolute Error (MAE) were used to evaluate the reliability of the models.


$$R^{2}=1-[\frac{\Sigma_{i=1}^{m}{\left(f_{i}-{\hat{f}}_{i}\right)}^{2}}{\Sigma_{i=1}^{N}{\left(f_{i}\right)}^{2}}]$$ (16)

$$RMSE=\sqrt{\frac{1}{m}\overset{m}{\underset{i=1}{\Sigma }}{\left(f_{i}-{\hat{f}}_{i}\right)}^{2}}$$ (17)

$$MAE=\frac{1}{m}\overset{m}{\underset{i=1}{\Sigma }}|f_{i}-{\hat{f}}_{i}|$$ (18)

### 3.8 Approach flowchart

The schematic diagram of the research consists of 4 steps:

- The data is randomly split into training datasets of 70% and test datasets of the remaining 30% of data. The training data is used to train the models Random Forest (RF), Artificial Neural Network (ANN), Gradient Boosting (GB), Extreme Gradient Boosting (XGB).
- PSO and K-fold cross-validation algorithms are used to select the optimal hyperparameters. The measure to select the set of hyperparameters for optimal performance is the coefficient of determination (R2).
- The models are compared, and the prediction results and standard deviations are measured through the coefficients of determination (R2), the root mean square error (RMSE), and the mean absolute error (MAE).
- The models with the best predictive results and standard deviations are used to observe the influence of the variables on the model’s performance.

**Fig 3:**
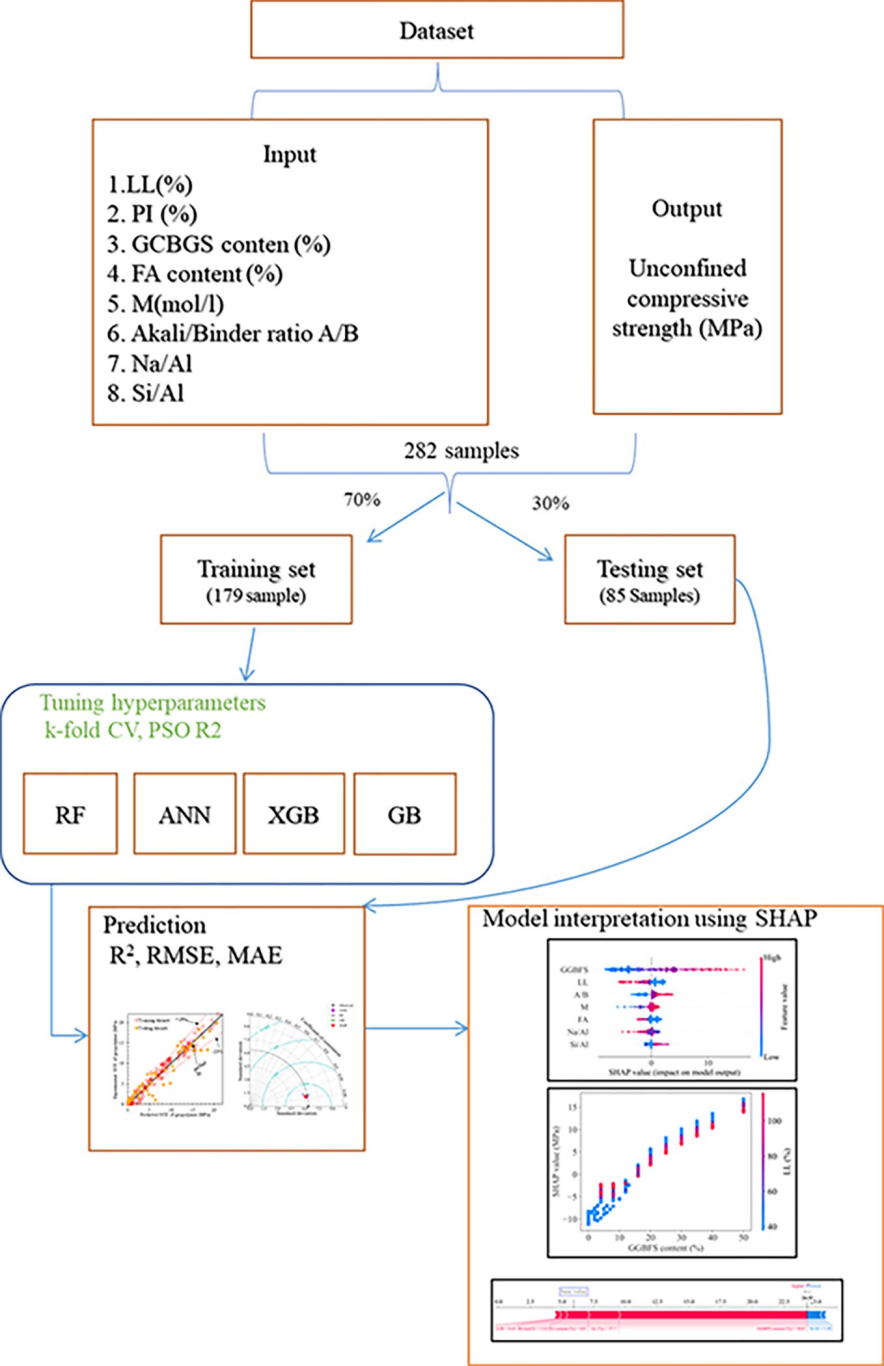
Methodology of this study.

The schematic diagram is shown as Fig 3.

## 4. Results and discussion

### 4.1 Determining optimal hyperparameters of machine learning models

Each solver uses a distinctive method to locate the finest outcome, and none of these algorithms is superior to the others. Without trying them all, it is challenging to determine which solver works best on a dataset. The ideal hyperparameters are debatable and unique for each dataset. The primary idea behind hyperparameter optimization is that the only method to discover the best hyperparameters for a dataset is through trial and error. To determine which resulting subgroup performs the best on a specific dataset, hyperparameter optimization examines a range of values.

The optimized parameters include max depth of the tree(max_depth), the number of trees (n_estimators), the learning rate of the tree (learning_rate), and minimum loss reduction required to make a further partition on a leaf node of the tree (min_split_loss). Max depth has the effect of avoiding overload, the number of n-estimators will affect the time and accuracy of the model. The meaningful learning rate avoids overfitting. Because of their important meanings, these parameters need to be optimized for the model to have the best possible performance. The search space for XGB’s parameters is as follows: number of trees from 10 to 200, learning rate from 0.001 to 0.9, max depth from 1 to 5 and min split loss from 1 to 5.

PSO and K-Fold CV algorithms are used to tune the hyperparameters. The hyperparameters are selected based on the R^2^ index between the predicted value and the experimental value. In this study, inertia, cognitive weight, social weight, temp weight, and rand rest were selected as inertia = 0.4, cognitive weight = 0.7, social weight = 0.7, temp weight = 0.3, and rand_rest_p = 0.05.

**Fig 4:**
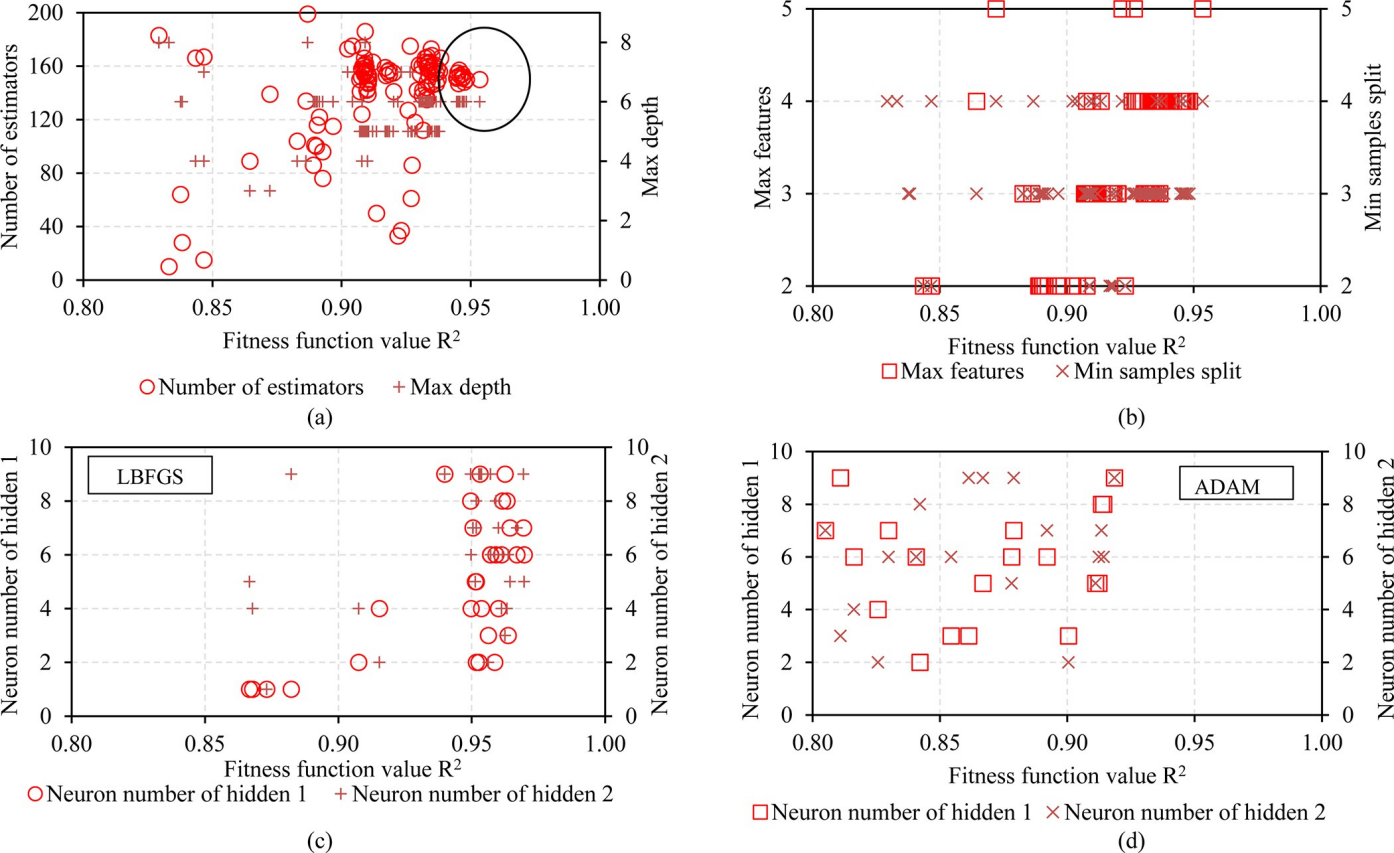
Influence of hyperparameter value on fitness function R^2^ value: (a) Number of estimations vs Max depth, (b) Max features vs Min samples split for RF model; (c) Neuron number of hidden 1 vs Neuron number of hidden 2 with solver LBFGS, (d) Neuron number of hidden 1 vs Neuron number of hidden 2 with solver ADAM for ANN model.

**Fig 5:**
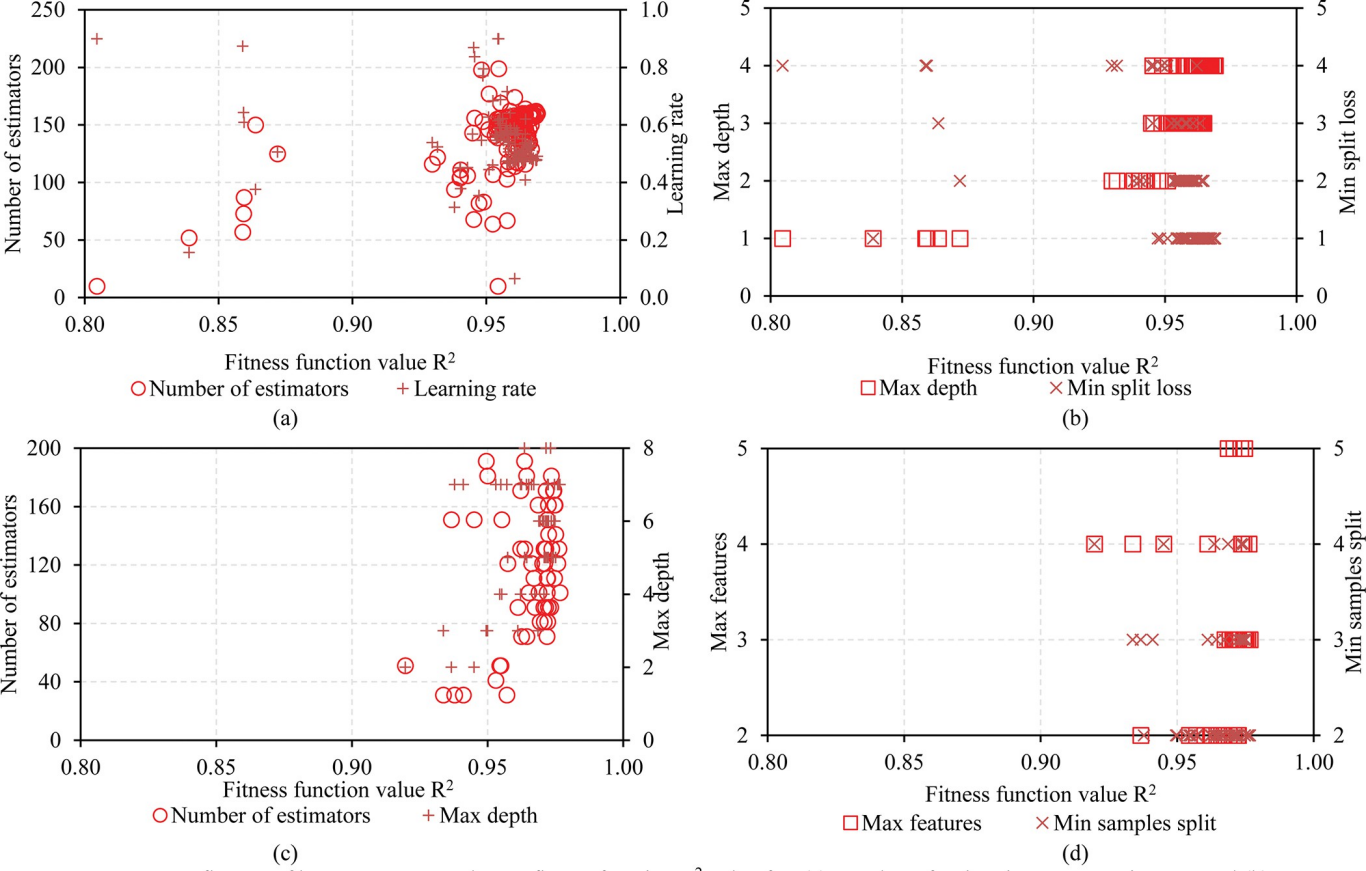
Influence of hyperparameter value on fitness function R^2^ value for: (a) Number of estimations vs Learning rate and (b) Max depth vs Min split loss for XGB model; (c) Number of estimations vs Max depth, (d) Max features vs Min samples split for GB model.

Figs 4 and 5 show the influence of hyperparameters on the volatility of the R^2^ metric, respectively. The vertical axis of the graph shows the values of the hyperparameters, the horizontal axis shows the value of the corresponding R^2^ metric. Fig 4A and 4B show the estimated values for the hyperparameters of the RF model. The best R^2^ value is 0.9535 when n-estimator is set to 150, max depth is 6, max features is 5, min samples split is 4, and min samples leaf is 2. Fig 4C and 4D represent price changes. R^2^ metric value of the ANN model with Limited-memory Broyden–Fletcher–Goldfarb–Shanno (LBTGS) optimization solver and adaptive moment estimation (ADAM). It is easy to see that the LBTGS solver has a slightly better R2 metric than the ADAM solver with R2 of LBTGS = 0.9696. Fig 5 shows the influence of the hyperparameters of the XGB and GB models on the R^2^ evaluation metric. It can be seen that the R2 value of the XGB model does not change significantly when the learning rate is set to 0.5 and the n-estimators is set to 150 (Fig 5A). In Fig 4B the R2 value increases quite uniformly when the max depth and min split loss values are set at 4. Looking at Fig 5C and 5D, there is no obvious trend for each hyperparameter of the GB model. Table 4 details the selected optimal sets of hyperparameters of the models for prediction.

**Table 4:** Optimal hyperparameters of ML models.

ML model	Optimal hyperparameters	Value	Fitness function R^2^ value
RF	Number of estimators	150	0.9535
	Max depth	6	
	Max features	5	
	Min samples split	4	
	Min samples leaf	2	
ANN	Activation function	Relu	0.9696
	Solver	LBFGS	
	Neuron number of hidden 1	6	
	Neuron number of hidden 1	5	
	Maximum iteration number	12000	
XGB	Number of estimators	160	0.9690
	Max depth	4	
	Learning rate	0.491	
	Min split loss	1	
GB	Number of estimators	101	0.9767
	Max depth	7	
	Max features	3	
	Min samples split	2	
	Min samples leaf	3	

### 4.2 Prediction of unconfined compressive strength based on ML models

**Fig 6:**
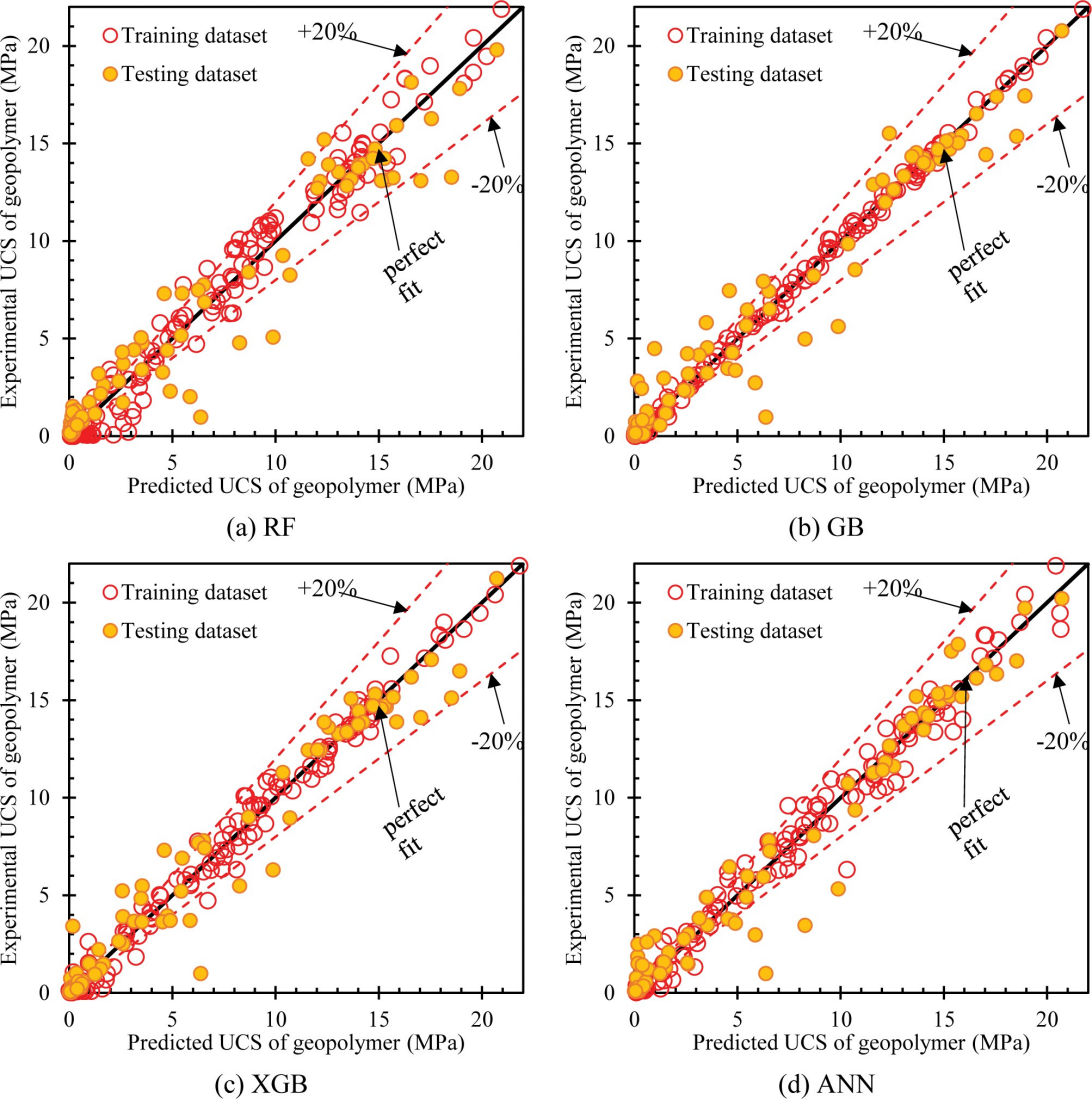
Experimental UCS of geopolymer vs predicted UCS based on (a) RF model, (b) GB model, (c) XGB model and (d) ANN model.

After selecting the optimal set of hyperparameters, the models make predictions and are evaluated for accuracy by the R^2^, RMSE, and MAE indices. Correlation between experimental values and predicted values. The prediction is shown in Fig 6. The RF model is a linear regression model with high accuracy, but the results show that its performance is not very good, with measurement indexes on the training set of 0.9824, RMSE of 0.8665 MPa, and MAE of 0.6051 MPa; these scores on the test set also give the weakest results among the four models, with R^2^ of 0.9459, RMSE of 1.4795, and MAE of 0.9706, as shown in Fig 6A. Looking at Fig 6D, it is easy to see that the ANN model has the best results, with the prediction points on the two datasets lying relatively compactly within +20% of the perfect fit. The XGB model is known for its high performance in the classification problem, as shown in Fig 6C, this model has good performance in the regression prediction problem. The evaluation results of the GB model in Fig 6B are relatively high for the number of data points with deviations beyond the +20% line. Table 5 summarizes the evaluation results based on the evaluation criteria R^2^, RMSE, and MAE on the two training and testing datasets.

**Table 5:** Performance value including R2, RMSE and MAE of ML models.

Model		Training dataset			Testing dataset	
	R^2^	RMSE (MPa)	MAE (MPa)	R^2^	RMSE (MPa)	MAE (MPa)
ANN	0.9883	0.7084	0.4311	0.9676	1.1445	0.7140
RF	0.9824	0.8665	0.6051	0.9459	1.4795	0.9706
XGB	0.9929	0.5500	0.3767	0.9671	1.1537	0.7357
GB	0.9985	0.2543	0.1779	0.9604	1.2660	0.7947

**Fig 7:**
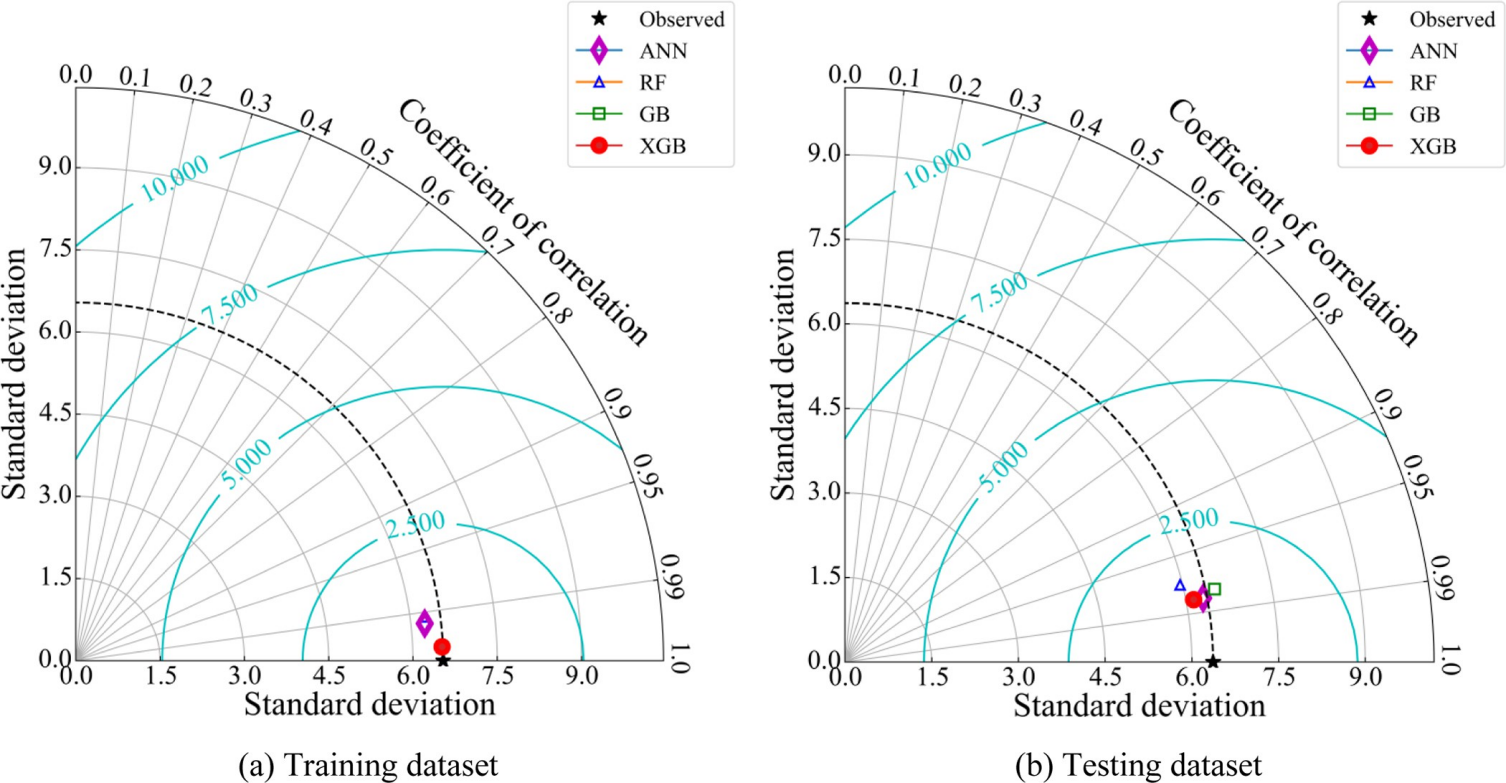
Taylor diagram for comparing UCS predicted by ML models and experimental UCS of geopolymer in (a) training dataset and (b) Testing dataset.

The performance of the four predictive models is further evaluated by the Taylor diagram in Fig 7. On the training set, it can be seen that the XGB model has the closest prediction to the experimental value. However, on the test dataset, the ANN model was slightly better than the XGB model. The RF and GB models have relatively good performance, but the scores for the ANN and XGB models are closer to the experimental values than the RF and GB models. Through the above analysis, it can be seen that ANN and XGB models have better performance than RF and GB models. The models can be arranged in order of optimal performance as follows: ANN > XGB > GB > RF.

### 4.3 SHAP value in interpretating of four machine learning models

Game theory concepts serve as the foundation for Shapley additive explanations (Shapley Value or SHAP). This allows a prediction to be described by supposing that each feature value of the instance is a "player" in a game. By including and excluding each player from all subsets of the other players, their contribution is assessed. The Shapley Value of a player is the weighted sum of their contributions. Shapley Value may be characterized as local precision and addition. Adding the Shapley Values of each feature to the base value, which represents the prediction average, yields the precise prediction value.

The SHAP technique [28] is used to examine model results in order to better understand the predictions provided by the high-performance ML model as well as to disclose the impact of each feature on the compressive strength of geopolymer concrete, which consists of both a global and local impact. It should be mentioned that this idea might be used for various machine learning-based prediction models. The game theory-derived additive feature attribution technique SHAP adds the input variables linearly as its output. The so-called Shapley value [29] serves as a representation of each feature’s contribution. As a result, the explanatory model *CON*(*VEC*) may be described as

$$CON(VEC)=\phi_{0}+\sum_{i=1}^{M}\phi_{i}VEC_{i}$$ (19)

where *VEC*, the dataset’s original input variables, were condensed into a vector of input variables, and M is the number of features in the data set, *ϕ*_0_ is the default constant value and reflects the attribution values for each feature when all inputs are null. The book [30] goes into further information about SHAP value idea.

**Fig 8:**
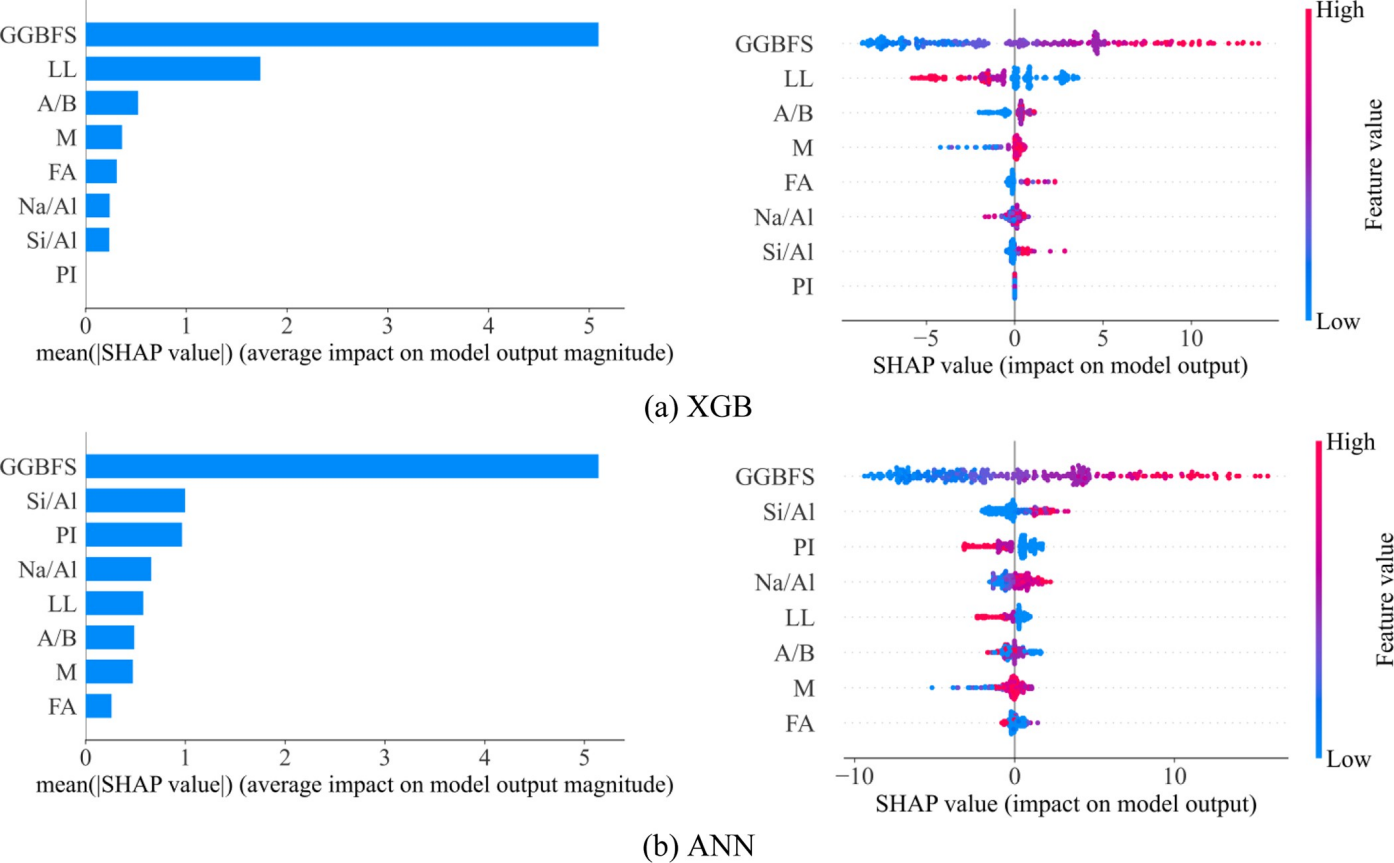
Global interpretation of two best ML model-based Shapley additive explanations.

Two models have the best performance ANN and XGB, the importance of input parameters is evaluated by the SHAP method. Fig 8A ranks the importance of the various input variables to the UCS output. The input variables are ranked according to their influence: GGBFS > LL > A/B > M > FA > Na/Al > Si/Al > PI. Notably, the feature with the least influence, PI, with the highest absolute contribution is relative equal to zero; thus, for a new database, the XGB model and the ANN model used features such as GGBFS, LL, A/B, M, FA, Na/Al, and Si/Al to build new database. The new performance values of the XGB model and the ANN model are shown in Table 6. Based on the new database, the performance of the XGB model is unchanged, while the performance of the ANN model is strongly improved.

**Table 6:** New performance values value including R^2^, RMSE and MAE of XGB and ANN models.

Model		Training dataset			Testing dataset	
	R^2^	RMSE (MPa)	MAE (MPa)	R^2^	RMSE (MPa)	MAE (MPa)
ANN	0.9917	0.5943	0.3787	0.9808	0.8808	0.6344
XGB	0.9929	0.5500	0.3767	0.9671	1.1537	0.7357

**Table 7:** Comparison of performance values and database between this study and other investigation of literature.

Reference of investigation	Number of data samples	Number of input variables	Performance metrics
Mozumder and Laskar (2015) [12]	282	8 inputs LL, PI, GGBFS, FA, M, A/B Na/Al, Si/Al	R^2^ = 0.9643 for testing dataset
Mozumder et al. (2017) [13]	213	7 inputs LL, PI, GGBFS, M, A/B Na/Al, Si/Al	R^2^ = 0.9801 for testing dataset
Javdanian and Lee (2019) [14]	282	8 inputs LL, PI, GGBFS, FA, M, A/B Na/Al, Si/Al	R^2^ = 0.9710 RMSE = 0.4010 MPa MAE = 0.2310 MPa for testing dataset
Soleimani et al. (2018) [16]	282	8 inputs LL, PI, GGBFS, FA, M, A/B Na/Al, Si/Al	R^2^ = 0.9420 MAE = 1.0710 MPa for testing dataset
Zeini et al. (2023) [17]	282	8 inputs LL, PI, GGBFS, FA, M, A/B Na/Al, Si/Al	R^2^ = 0.9757 RMSE = 0.9815 MPa
This study	282	7 inputs LL, GGBFS, FA, M, A/B Na/Al, Si/Al	ANN model for testing dataset R2 = 0.9808 RMSE = 0.8808 MPa MAE = 0.6344 MPa

**Fig 9:**
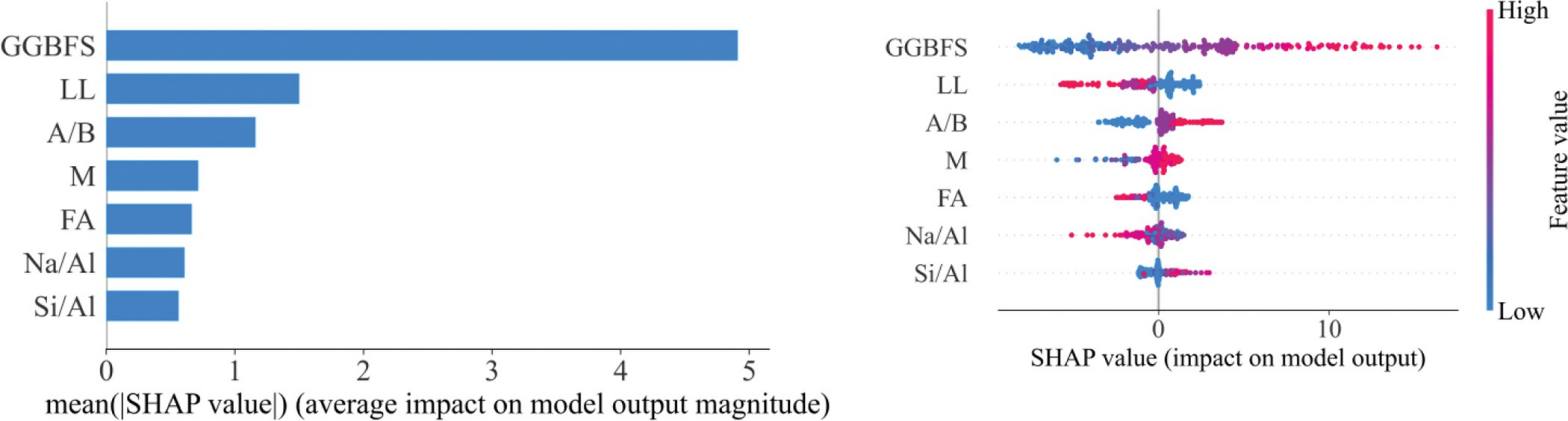
Global interpretation of ANN model-based Shapley additive explanations using the new database.

The ANN model after using the new dataset has a change in the order of importance of the features compared to when the PI feature was still there; there is a change in the influence ranking of the features. Previously, when the dataset included the PI feature, the ranking was GGGFS > Si/Al > PI > Na/Al > LL > A/B > M > FA (Fig 8B). Then, when removing the PI feature from the dataset, the new rating is similar to that of the model XGB GGBFS > LL > A/B > M > FA > Na/Al > Si/Al observed in Fig 9.

In particular, Table 7 shows a comparison of performance values and database between this study and other investigation of literature summarized in the introduction section. It is worth noting that the new database without input variable Plasticity index (PI) can enhance strongly the performance of ANN model in comparing with other ML model proposed in the literature for predicting UCS of geopolymer stabilized soil.

**Fig 10:**
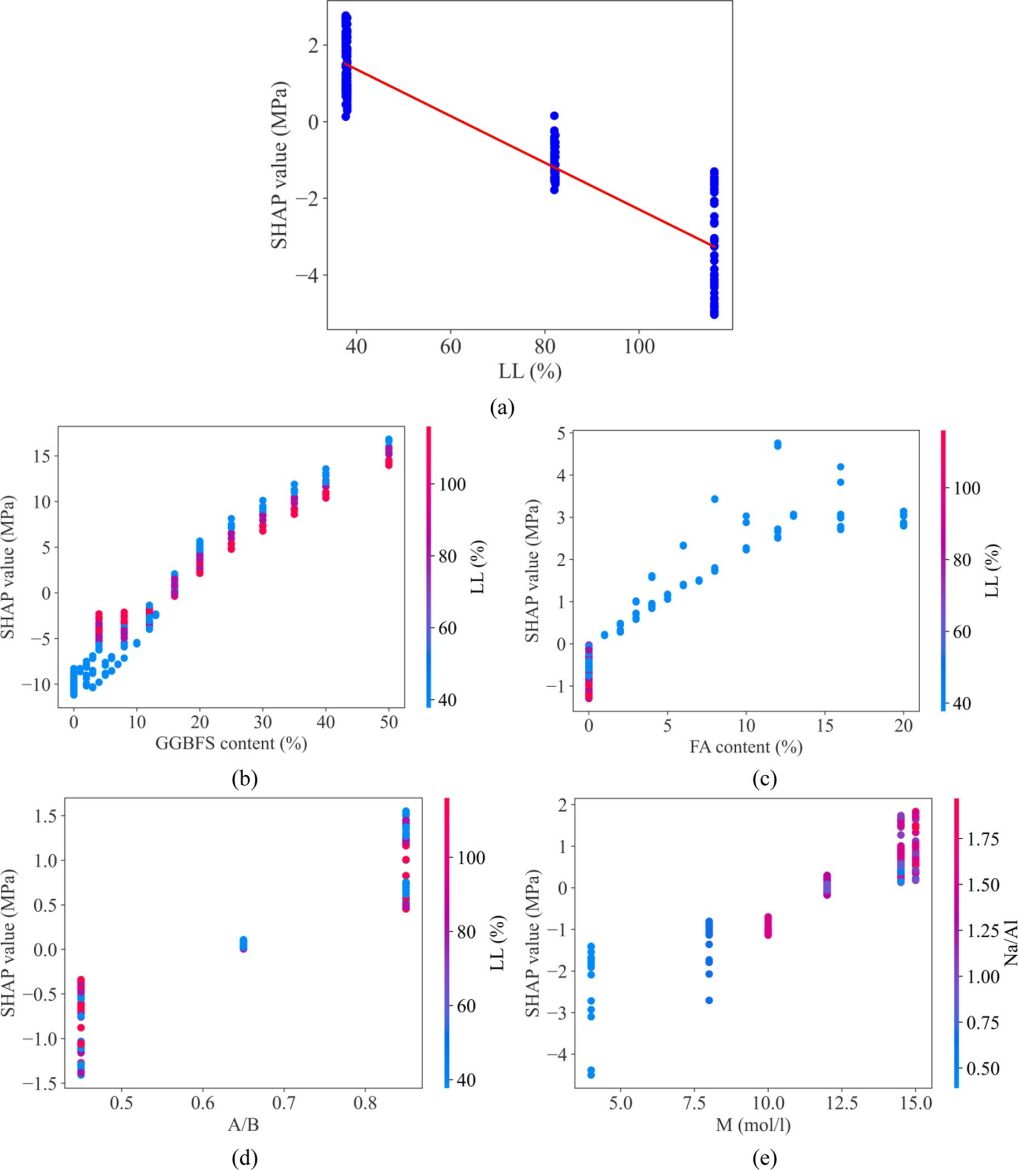
Local interpretation of ANN model-based Shapley additive explanations using the new database.

Fig 10A shows a clearly demonstrated negative relationship between the LL feature and the output value. Through Fig 10, it can be seen that as GGBFS content, FA content, A/B, and M increase, the SHAP value also increases. This suggests that they are positively correlated with UCS and may reduce the effect of the negative correlation between LL and UCS. In particular, when observing Fig 10B, it can be seen that despite the negative relationship between LL and UCS, when increasing the GGBFS content, the SHAP value also increases rapidly. In addition, explanations for the contributions of all input variables are provided in three specific cases.

**Fig 11:**
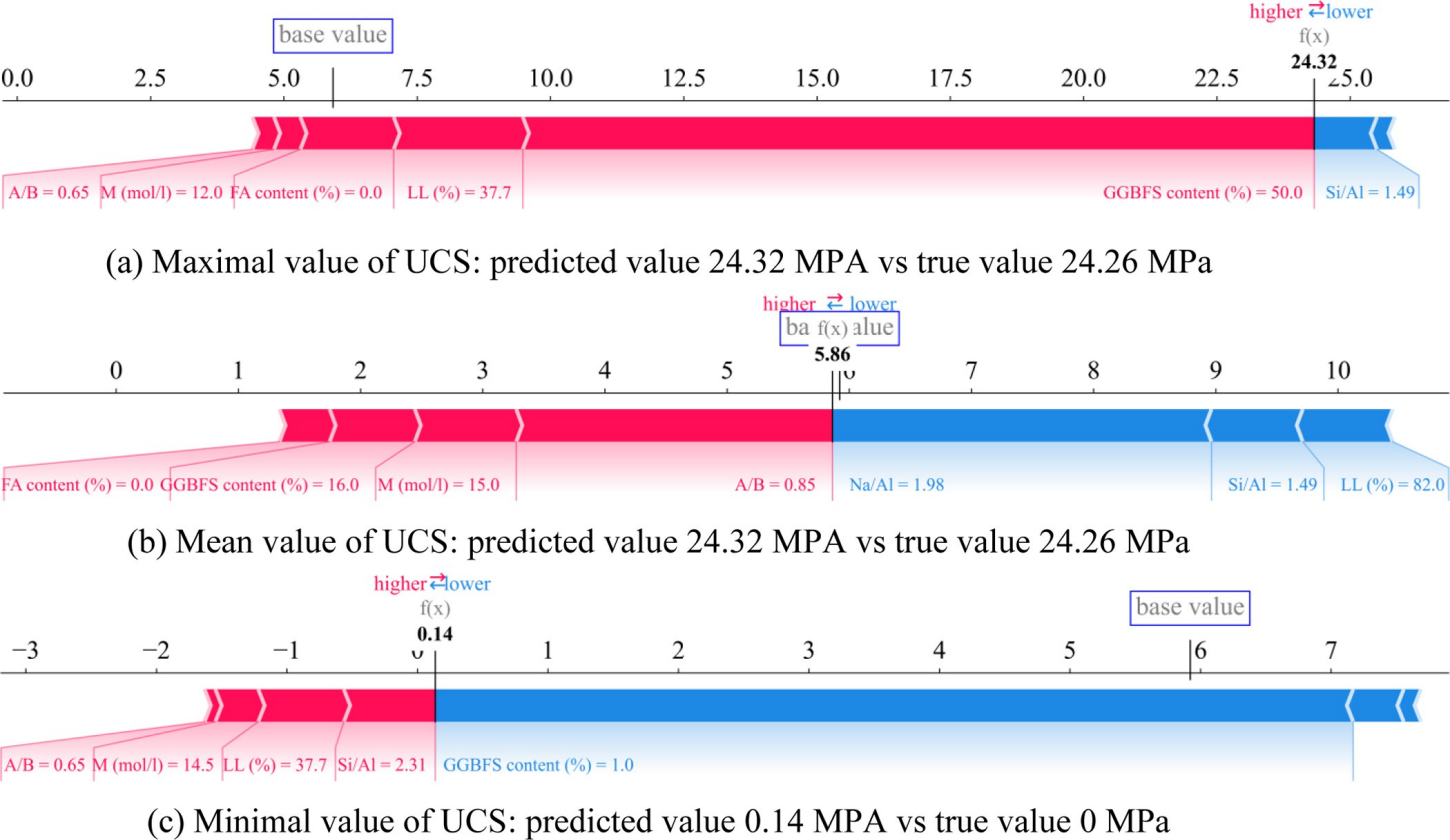
Individual interpretation of ANN model-based Shapley additive explanations using the new database for three specific cases.

The mean value on the new dataset that has removed the PI feature in cases 1, 2, and 3 in Fig 11A-11C is 5.86 MPa. Sample 1 has typical positive features of A/B, M, FA content, LL, and GGBFS content and typical negative features of Si/Al. Combining the Shap value of the features and the base value, the predicted value is 24.23 MPa, which is quite close to the actual value of 24.26 MPa. For case 2, the features that contribute to UCS enhancement are FA content, GGBFS content, M, and A/B. Meanwhile, features leading to reduction include Na/Al, Si/Al, and LL. The final prediction is pushed to 24.23 MPa, which is near the actual value of 24.26 MPa when the base value and the SHAP values for the features are combined. Similar to case 3, positive features include A/B, M, LL, and Si/Al. The feature that is inversely correlated with UCS is GGBFS content. Combining the Shap values of the features and the base value, the predicted value is 0.14 MPa, which is relatively close to the true value of 0 MPa. Thereby, it can be seen that the influence of GGBFS content has a very strong positive correlation, whereas LL (%) has a negative correlation with the growth of UCS.

## 5. Conclusions

In this study, methods for RF, ANN, XGB, and GB were developed to forecast the unconfined compressive strength of cohesive soils stabilized with geopolymer. To create databases and create models, 282 experimental findings were gathered. 70% of the data in this database were arbitrarily chosen for the training phase, and the other 30% were used for the testing phase. The research demonstrates that the ANN model is the most reliable and yields the best prediction performance. For the test set, the best model’s R^2^, RMSE, and MAE values are, respectively, 0.9808, 0.8808 MPa and 0.6344 MPa. In this research, the dependence of the eight input variables on the predicted outcomes was examined using the Shap summary plot interpretation analysis. GGBFS content was found to be the most significant factor affecting the unconfined compressive strength of cohesive soils stabilized with geopolymer based on the findings of the XGB model, while PI was found to be the least significant factor. The GGBFS content and the LL are the two main influencing parameters, according to the results of using the ANN model after removing the feature that has the least impact on the prediction to build a new dataset.

Based on SHAP value, the order of feature effect can be sorted in descending as GGBFS > LL > A/B > M > FA > Na/Al > Si/Al. Using these seven inputs, the ANN model can achieve the highest accuracy. Where GGBFS has a positive relationship with the development of unconfined compressive strength, LL has a negative relationship. The findings of this study can be used to create a trustworthy soft computational instrument for timely and quick prediction of the unconfined compressive strength of cohesive soils stabilized with geopolymer. The prediction method can then shorten the duration and lower the cost of experiments.

## Supporting information

S1 Data (CSV)
